# Consensus statement on the transition process from pediatric care to adult care in patients with chronic inflammatory rheumatic diseases with childhood-onset

**DOI:** 10.1186/1546-0096-12-S1-P85

**Published:** 2014-09-17

**Authors:** Inmaculada Calvo, Jordi Anton, Sagrario Bustabad, Marisol Camacho, Jaime De Inocencio, Mª Luz Gamir, Genaro Graña, Lucía La Cruz, Juan Carlos López-Robledillo, Marta Medrano, Rosa Merino, Consuelo Modesto, Esmeralda Nuñez, Mª Jesus Rua, Vicent Torrente, Carmen Vargas, Estibaliz Loza

**Affiliations:** 1 Hospital La Fe , Valencia, Spain; 2 Hospital Sant Joan de Deu, Esplugues de Llobregat, Spain; 3 Hospital Universitario de Canarias, Santa Cruz de Tenerife, Spain; 4 Hospital Universitario Virgen del Rocío, Sevilla, Spain; 5 Hospital 12 de Octubre, Madrid, Spain; 6 Hospital Ramón y Cajal, Madrid, Spain; 7 Complejo Hospitalario Universitario A Coruña, La Coruña, Spain; 8 Hospital Universitari Son Dureta, Mallorca, Spain; 9 Hospital del Niño Jesús, Madrid, Spain; 10 Hospital Universitario Miguel Servet, Zaragoza, Spain; 11 Hospital La Paz, Madrid, Spain; 12 Hospital Vall d'Hebron, Barcelona, Spain; 13 Hospital Regional Universitario Carlos Haya, Malaga, Spain; 14 Hospital Universitario Cruces, Barakaldo, Spain; 15 Hospital Sant Joan de Déu, Esplugues de Llobregat, Spain; 16 Hospital Universitario Virgen de la Macarena, Sevilla, Spain; 17 Institute for Musculoskeletal health, Madrid, Spain

## Introduction

Many young people with childhood-onset diseases, including rheumatic diseases, continue to require medical care into adult life. Although there is an extensive evidence base for the need of transitional care, there is a paucity of robust outcome data and a great variability on the models of transitional care.

## Objectives

To develop recommendations on the transition from pediatric care to adult care in patients with chronic inflammatory rheumatic diseases with childhood-onset based on the best evidence and experience.

## Methods

Recommendations were generated following nominal group methodology and Delphi technique. A panel of experts was established (8 pediatricians, 8 rheumatologists). A systematic literature review (on transitional care) and a narrative review (websites, clinical guidelines and other relevant documentation) were performed and presented to the panel in the 1^st^ panel meeting to be discussed and to help define recommendations. A first draft of recommendations was generated and circulated for comments and wording refinements. Focal groups with adolescents, young adults and parents were separately. In a 2^nd^ panel meeting the focus group results along with the input from invited nurses and psychologists were used to established definitive recommendations. Then, a Delphi process (2 rounds) was carried out. A large group of 70 pediatricians and rheumatologists took part. Recommendations were voted from 1 (total disagreement) to 10 (total agreement). We defined agreement if at least 70% voted ≥7. The level of evidence and grade or recommendation was assessed using the Oxford Centre for Evidence-based Medicine Levels of Evidence.

## Results

Transition care was defined as a purposeful, planned process that addresses the medical, psychosocial and educational/vocational needs of adolescents and young adults with chronic inflammatory rheumatic diseases with childhood-onset as they move from child-centred to adult-oriented health care systems. The consensus covers: transition needs, barriers and facilitators, transitional issues (objectives, participants, content, phases, timing, plans, documentation, and responsibilities), physicians and other health professionals knowledge and skills requirements, models/programs, strategies and guideline for implementation. A total of 19 recommendations were developed.

## Conclusion

These recommendations are intended to provide pediatricians, rheumatologists, patients, families and other stakeholders with a consensus on the transition process from pediatric care to adult care in patients with chronic inflammatory rheumatic diseases with childhood-onset.

## Disclosure of interest

I. Calvo: None declared., J. Anton: None declared., S. Bustabad: None declared., M. Camacho: None declared., J. De Inocencio: None declared., M. L. Gamir: None declared., G. Graña: None declared., L. La Cruz: None declared., J. C. López-Robledillo: None declared., M. Medrano: None declared., R. Merino: None declared., C. Modesto: None declared., E. Nuñez: None declared., M. J. Rua: None declared., V. Torrente: None declared., C. Vargas: None declared., E. Loza Grant / Research Support from: Abbvie.

